# Comparative study of CEA and CA19-9 in esophageal, gastric and colon cancers individually and in combination (ROC curve analysis)

**DOI:** 10.7497/j.issn.2095-3941.2013.03.005

**Published:** 2013-09

**Authors:** Bhawna Bagaria, Sadhna Sood, Rameshwaram Sharma, Soniya Lalwani

**Affiliations:** 1 Department of Biochemistry, S.M.S. Medical College & Hospital, Jaipur 302004, India;; 2 Department of Radiotherapy and Oncology, S.M.S. Medical College & Hospital, Jaipur 302004, India;; 3 Advanced Bioinformatics Centre, Birla Institute of Scientific Research, Jaipur 302004, India

**Keywords:** Carcinoembryonic antigen, carbohydrate antigen 19-9, human, Receiver operating characteristic curve, sensitivity and specificity

## Abstract

**Objective:**

To determine the clinical serum levels of carcinoembryonic antigen (CEA) and carbohydrate antigen 19-9 (CA19-9), individually and in combination, for the diagnosis of 50 healthy subjects and 150 cases of esophageal, gastric, and colon cancers.

**Methods:**

The sensitivities of the two markers were compared individually and in combination, with specificity set at 100%. Receiver operating characteristic (ROC) curves were plotted.

**Results:**

Serum CEA levels were significantly higher in cancer patients than in the control group. The sensitivity of CEA was determined: in esophageal cancer, sensitivity=28%, negative predictive value (NPV)=61.72%, and AUC=0.742  (SE=0.05), with a significance level of *P*<0.0001; in gastric cancer, sensitivity=30%, NPV=58.82%, and AUC=0.734 (SE=0.05), with a significance level of *P*<0.0001; in colon cancer, sensitivity=74%, NPV=79.36%, and AUC=0.856  (SE=0.04), with a significance level of *P*<0.0001. The sensitivity of CA19-9 was also evaluated: in esophageal cancer, sensitivity=18%, NPV=54.94%, and AUC=0.573 (SE =0.05), with a significance level of *P*=0.2054. In gastric cancer, sensitivity=42%, NPV=63.29%, and AUC=0.679 (SE =0.05), with a significance level of *P*<0.0011. In colon cancer, sensitivity=26%, NPV=57.47%, and AUC=0.580 (SE =0.05), with a significance level of *P*=0.1670. The following were the sensitivities of CEA/CA19-9 combined: in esophageal cancer, sensitivity=42%, NPV=63.29%, SE=0.078 (95% CI: 0.0159-0.322); gastric cancer, sensitivity=58%, NPV=70.42%, SE=0.072 (95% CI: -0.0866-0.198); and colon cancer, sensitivity=72%, NPV=78.12%, SE=0.070 (95% CI: 0.137-0.415).

**Conclusion:**

CEA exhibited the highest sensitivity for colon cancer, and CA19-9 exhibited the highest sensitivity for gastric cancer. Combined analysis indicated an increase in diagnostic sensitivity in esophageal and gastric cancer compared with that in colon cancer.

## Introduction

Cancer mortality and morbidity have increased in recent years, with gastrointestinal cancers comprising the majority of overall malignant conditions. All Indian cancer registries identify the digestive system as the most common cancer site in males. In women, breast cancer exhibits the highest occurrence, followed by cancers of the genital organs and the digestive system^1^. The incidence of digestive cancer, including cancer of the esophagus, stomach, colon, and liver, is analyzed in developing and less developed countries in Africa, Asia, the Caribbean, and Latin America. The analysis is based on cancer registries for observed values, published at International Agency for Research on Cancer and on the GLOBOCAN 2008 database for estimations. A low survival rate, even for localized cases, suggests severe deficiencies in early diagnosis and effective treatment in India^2^. A strategy to control the disease is based on promoting awareness of risk factors for cancer while maintaining a traditional lifestyle, as well as investing in early diagnosis and adequate treatment^3^.

Tumor markers are potentially useful in early diagnosis of cancer. They are produced as biologically active substances in the tumor tissue or cancer cells because of abnormal expression of genes. These substances are either not produced or produced in very small amounts in normal tissues and benign lesions^4^. Carcinoembryonic antigen (CEA), originally described by Gold and Freedman in 1965, is currently classified under the immunoglobulin super family and functions as an intracellular adhesion molecule. CEA is a glycosylphosphatidylinositol-cell surface anchored glycoprotein with specialized sialofucosylated glycoforms that act as functional colon carcinoma L-selectin and E-selectin ligands, which may significantly affect the metastatic dissemination of colon carcinoma^5^^,^^6^. Serum samples from individuals with colorectal carcinoma, gastric carcinoma, pancreatic carcinoma, lung carcinoma, and breast carcinoma, as well as those with medullary thyroid carcinoma, exhibited higher levels of CEA than did those from healthy individuals^7^.

Carbohydrate antigen 19-9 (CA19-9) is a type of glycosphingolipid that is a specific sialyzed derivative of the Lea blood group and shown as Lexa. The CA19-9 antigen was first isolated by Koprowski *et al.* in 1979 by using the monoclonal antibody 1116-NS-19-9 generated against colonic carcinoma cell lines. Subsequently, radioimmunometric assay was developed by Del Villano in 1983 to quantify it^8^. In plasma, it exists as a high-molecular-weight mucin glycoprotein containing the sialyated Lewis-a-epitope lacto-N fucopentose II. Recent reports indicated that serum CA19-9 is frequently elevated in subjects with various gastrointestinal malignancies, such as pancreatic, colorectal, gastric, and hepatic carcinomas^9^.

A promising technique to overcome the insensitivity of a single tumor marker is the simultaneous assay of several markers, given that cancer cells are biochemically heterogeneous and may synthesize a broad spectrum of tumor markers. Performing a series of assays can prevent missing a potential cell marker.

Therefore, the present study aims to compare CEA and CA19-9 in esophageal, gastric, and colon cancers, to evaluate the sensitivities of the two markers individually and in combination by analyzing their ROC curves before starting any treatment, and to determine whether the combined use of these markers could improve the diagnostic sensitivity in esophageal, gastric, and colon cancers.

## Material and methods

### Clinical data

The subjects included 150 patients suffering from esophageal, gastric, and colon cancer diagnosed by endoscopic examination and biopsy and who have not previously received any anticancer therapy. Fifty healthy subjects with no cancer comprised the normal control group.

The hematological and biochemical profile of each cancer patient and each healthy subject was evaluated. All patients and healthy control subjects were recruited from the Department of Radiotherapy, SMS Medical College and Hospital, Jaipur from July 2011 to December 2012. This study was approved by the Ethics Committee and the institutional research committee of the hospital. Written informed consent was obtained from all patients and healthy subjects.

### Inclusion criteria

Healthy subjects were identified as individuals not suffering from any physical ailment or acute illness, not hospitalized for any disease in the past two years, and not addicted to smoking, tobacco, or alcohol consumption.

Patients were identified as individuals suffering from esophageal, gastric, and colon cancers currently diagnosed by endoscopic examination and biopsy and who have not previously received any anticancer therapy.

### Exclusion criteria

Healthy subjects with any type of gastrointestinal infections, acute illness, recent hospitalization, or addiction to smoking, alcohol, or tobacco are excluded from this study. Cancer patients who have received radiotherapy, chemotherapy or surgery were excluded.

### Study design

#### Clinical history

Each patient was first examined by obtaining a brief clinical history related to diet, lifestyle, initial symptoms, or any previously received treatment.

The patients and healthy subjects were categorized as follows: Group 1: 50 normal healthy subjects; Group 2: 50 patients with esophageal cancer; Group 3: 50 patients with gastric cancer; Group 4: 50 patients with colon cancer.

#### Sample Collection

Blood samples were collected prior to administering any therapy in gastrointestinal cancer patients and as part of a routine investigation in healthy subjects. The samples placed in a plain vial were allowed to clot. Serum was separated by centrifugation at 3,485 *g* for 10 min and stored at –20 °C until further assay was performed.

CEA estimation was conducted using commercial IMMULITE-2000, a solid phase, two-site sequential chemiluminescent immunometric assay. IMMULITE-2000 Systems, SIEMENS HEALTH CARE DIAGNOSTICS PRODUCT LTD. L Lanberis, Gwynedd, LL554EL, UK Ref: L2KCE2; Lot: 273/2012-10. CA19-9 estimation was conducted using commercial calbiotech CA19-9 ELISA Kit, based on solid phase enzyme-linked immunosorbent assay. CA19-9 ELISA kit 96 T (CalBiotech, USA), CATALOG NO: RN-42627/2012-11.

The tests were performed strictly according to the manufacturer’s instructions and as stated in the literature. Frequent false-positive outcomes result from benign gastro-intestinal disorders and smoking. Thus, the threshold values for CEA in GI cancers according to the kit were as follows: male smokers: 6.2 ng/mL; male nonsmokers: 3.4 ng/mL; female smokers: 4.9 ng/mL; female nonsmokers: 2.5 ng/mL; healthy men and women: CA19-9 assay values below 35 U/mL.

## Results

### Data analysis

Data were analyzed using SPSS version 10.0 (SPSS Inc., USA) and MedCalc to estimate the significance of the observed differences, calculate sensitivity, and negative predictive value (NPV) (with specificity at 100%). ROC curves were plotted.

No significant differences in gender and age were indicated between the cancer groups and the healthy control subjects (*P*>0.05) (**Table 1**). The mean serum levels of the tumor markers were shown in **Table 2**. The mean serum level of CEA in patients of esophageal, gastric, and colon cancers were significantly higher than in healthy subjects. The overall mean serum level of CA19-9 in patients with esophageal cancer was not significantly higher than that of the control group, except for a few cases. These cases showed high CA19-9 levels attributed to the advanced stage of cancer. The mean serum levels of CA19-9 were significantly higher in gastric and colon cancers than in healthy control subjects. The range of CA19-9 in gastric cancer patients (6.07-80.546) U/mL and in colon cancer patients (6.078-98.67) U/mL was considerably higher as compared to normal range (below 35 U/mL).

**Table 1:** Comparison of gender and age of esophageal, gastric and colon cancer patients with healthy control subjects

Group	*n*	M/F*	Comparison of gender		Age, mean±SD (range)	Comparison of age	
			*χ*^2^ (df)	*P*		*t*	*P*
Control	50	25/25			47.54±10.73 (29-72)		
Esophagus cancer	50	28/22	0.36	0.54	50.88±8.17 (26-65)	1.7509	0.0831
Gastric cancer	50	34/16	3.35	0.06	48.88±11.66 (21-68)	0.5978	0.5514
Colon cancer	50	30/20	1.01	0.31	43.24±12.69 (22-70)	1.8294	0.0704

*M: male; F: female

**Table 2:** Comparison of CEA and CA19-9 between gastrointestinal cancer patients and healthy control subjects before therapy

Group	CEA, mean±SD (range)	CA 19-9, mean±SD (range)
Control	2.23±0.82 (0.96-3.34)	17.18±8.49 (6.07-34.85)
Esophageal cancer	5.57±5.98* (1.11-28.70)	21.70±13.73 (7.17-71.27)
Gastric cancer	6.23±7.73* (1.00-30.00)	28.11±18.14* (6.07-80.55)
Colon cancer	12.94±13.47* (1.12-50.00)	25.52±21.59* (6.08-98.67)

*P<0.05.

In a receiver operating characteristic (ROC) curve, the true positive rate (sensitivity) is plotted as a function of the false positive rate (100% specificity) for different threshold values. Each point on the ROC curve represents a sensitivity/specificity pair corresponding to a particular decision threshold. A test with perfect discrimination (no overlap in the two distributions) has a ROC curve passing through the upper left corner (100% sensitivity, 100% specificity). Therefore, a ROC curve that is closer to the upper left corner indicates a higher overall accuracy of the test^10^.

**Figure 1:**
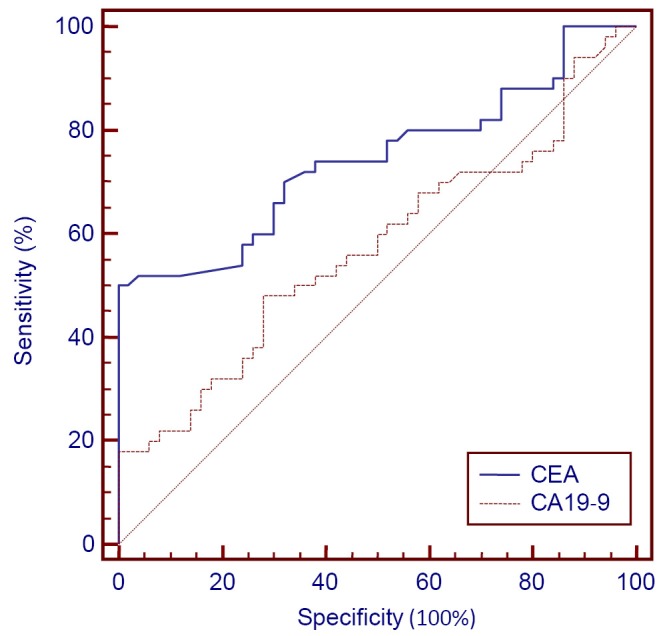
CEA+CA19-9 ROC in esophagus cancer.

**Figure 2:**
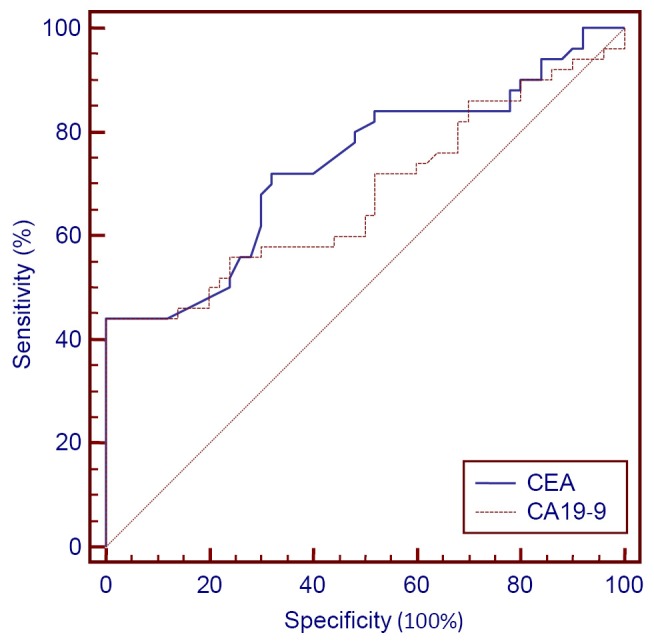
CEA+CA19-9 in gastric cancer.

**Figure 3:**
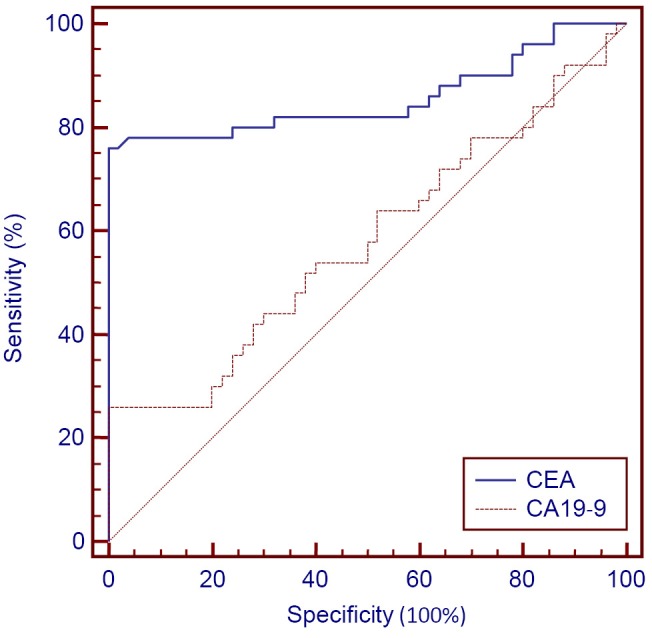
CEA+CA19-9 in colon cancer.

The sensitivity of CEA, CA19-9, and CEA/CA19-9, as well as the NPV for esophageal, gastric, and colon cancers were given in (**Table 3**, **Figures 1****,****2****,****3**). Serum CEA values exceed the threshold value in 38% of patients with esophageal cancer. NPV is 61.72%, AUC is 0.742 (SE=0.05), and the significance level is *P*<0.0001. CEA values exceed the threshold value in 30% of patients with gastric cancer. NPV is 58.82%, AUC is 0.734 (SE=0.05), and the significance level is *P*<0.0001. CEA values were higher than the threshold value in 74% of patients with colon cancer. NPV is 79.36%, AUC is 0.856 (SE=0.04), and the significance level is *P*<0.0001. The ideal threshold value identified from the ROC curve for CEA was >3.34 with a sensitivity of 50% (95% CI: 35.5-64.5) and a specificity of 100% in patients with esophageal cancer. The ideal threshold value based on the ROC curve for CEA was >3.34 with a sensitivity of 44% (95% CI: 30.0-58.7) and a specificity of 100% in patients with gastric cancer. The ideal threshold value based on the ROC curve for CEA was >3.34 with a sensitivity of 76% (95% CI: 61.8-86.9) and a specificity of 100% in patients with colon cancer.

**Table 3:** Effect on sensitivity on combined analysis of CEA and CA19-9 in esophagus, gastric and colon cancer

Tumor markers	Esophagus cancer			Gastric cancer			Colon cancer	
	Sensitivity (%)	NPV (%)		Sensitivity (%)	NPV (%)		Sensitivity (%)	NPV (%)
CEA	38	61.72		30	58.82		74	79.36
CA19-9	18	54.94		42	63.29		26	57.47
CEA/CA19-9	42	63.29		58	70.42		72	78.12

NPV, negative predictive value.

In this study, CA19-9 was above the threshold value in 18% of patients with esophageal cancer, with NPV of 54.94%, AUC of 0.573 (SE=0.05), and significance level of *P*=0.2054. CA19-9 exceeded the threshold value in 42% of patients with gastric cancer, with NPV of 63.29%, AUC of 0.679 (SE=0.05), and significance level of *P*<0.0011. CA19-9 was higher than the threshold value in 26% of patients with colon cancer, with NPV of 57.47%, AUC of 0.580 (SE=0.05), and significance level of *P*=0.1670. The ideal threshold point identified from the ROC curve for CA19-9 was >21.08, with a sensitivity of 48% (95% CI: 33.7-62.6) and a specificity of 72%. The ideal threshold point for CA19-9 was >34.851, with a sensitivity of 44% (95% CI: 30.0-58.7) and a specificity of 100% in patients with gastric cancer.

The ideal threshold point based on the ROC curve for CA19-9 was >34.851, with a sensitivity of 26% (95% CI: 14.6-40.3) and a specificity of 100% in patients with colon cancer.

Results from the ROC curve study showed that the ideal threshold point given by ROC curve for CEA in esophageal, gastric, and colon cancers was >3.34. For CA19-9 in esophagus cancer this value was >21.08, and for gastric and colon cancers, this value was >34.851.

Combined analysis of CEA/CA19-9 showed that sensitivity increased to 42%, with NPV of 63.29%, and SE of 0.078, (95% CI: 0.0159-0.322) in patients with esophageal cancer. In patients with gastric cancer, sensitivity increased to 58%, with NPV of 70.42% and SE of 0.072 (95% CI: -0.0866-0.198). In patients with colon cancer, sensitivity decreased to 72%, with NPV of 78.12% and SE of 0.070 (95% CI: 0.137-0.415).

## Discussion

Currently, no “universal” tumor marker that can detect any particular type of cancer has been identified. Mild elevation in a number of early-stage cancers have often been difficult to justify because many benign pathologies may cause such changes. An elevated tumor marker level may indicate cancer, however, this result fails to provide a sufficient basis for cancer diagnosis. Therefore, measurements of tumor markers are usually combined with other tests, such as biopsies.

In this study, the sensitivity of CEA detected in esophageal cancer was 38%. This result contradicts previous studies conducted by Mao *et al.*^11^ and Schneider *et al.*^12^ indicating CEA sensitivity of 29.1%, and 24%, respectively, in esophageal cancer which was lower than our result. Mao evaluated that patient with bulky or advanced tumor usually had much higher mean value than those with early stage tumors. The level of serum CEA varied greatly in a small part of the patients. Extremely elevated serum CEA usually indicated advanced lesion or tumor metastasis. Schneider suggested that for increasing sensitivity and/or specificity in gastro-intestinal cancer diagnosis, a combination of tumor markers is of certain interest. The results of the present study were in consistent with those obtained by Choudhary *et al.*^13^ who observed GIT carcinomas are common in younger age group i.e., below 30 and CEA is the sensitive test for the diagnosis of GIT carcinoma. Kosugi *et al.*^14^ also observed similar result as ours and suggested that serum CEA levels may be of use especially in predicting clinically inapparent distant metastasis. In gastric cancer, CEA sensitivity was 30%, which contradicts Hwang *et al.*^15^, results with preoperative CEA sensitivity of 15.4%. They observed the sensitivity was not sufficient, and found serial examinations to be useful in postoperative follow-up and detection of recurrence in patients with gastric cancer. Park *et al.*^16^ observed a CEA sensitivity 9.3% in gastric cancer patients they found that relatively higher cut-off level (7 ng/mL) seems to cause lower positive rate of the serum CEA (9.3%). The present study obtained results similar to those reported by Takahashi *et al.*^17^ They suggested that CEA and/or CA19-9 monitoring after operation was useful to predict the recurrence of gastric cancer, especially in almost all the patients with high preoperative levels of these markers. Mihmanli *et al.*^18^ observed elevating levels of CEA correlated with depth of invasion and pathological stage. Preoperative serum CEA and CA19-9 levels may add useful information in patients with gastric carcinoma, and CEA level is a predictor of prognosis. In the present study, the CEA sensitivity detected in patients with colon cancer was 74%. The CEA sensitivity reported by Carpelan- Holmstroma *et al.*^19^ was 54% and that by Palmqvist *et al.*^20^ was 12% in patients with colorectal cancer. They found although the specificity of the CEA test in its present form is high, the sensitivity is disappointingly low, prohibiting the use of the CEA test for mass screening. The findings of the present study were consistent with the studies by Bayatti *et al.*^21^ and Goldstein *et al.*^22^ They identified CEA as the most specific polysaccharide protein complex with a molecular weight of 22 kU and contributory to the malignant characteristics of the tumor. An elevated preoperative CEA is a poor prognostic sign and correlates with reduced overall survival after surgical resection of colorectal carcinoma. Frequent monitoring of CEA postoperatively may allow identification of patients with metastatic disease for whom surgical resection or other localized therapy might be potentially beneficial^22^. CEA is not usually present in the blood of healthy adults, although its levels are raised in heavy smokers. It is attached to the cell membrane by a glycosylphosphatidylinositol anchor and may be released as a soluble form by phospholipase C or phospholipase D. The structural similarity of CEA to certain immunoglobulin-related proteins, such as ICAM-1 and ICAM-2, initially suggested that CEA functions as an adhesion molecule. *In vitro* experiments showed that CEA was capable of both homophilic (CEA binding to CEA) and heterophilic (CEA binding to non-CEA molecules) interactions. CEA may significantly influence cancer invasion and metastasis because of the causal involvement of alterations in cell adhesion in these processes^23^.

The present study obtained a CA19-9 sensitivity of 18% in esophageal cancer patients, which is lower than that (34%) obtained by Mealy *et al.*^24^, but is consistent with the results obtained in previous cancer studies conducted in 2011^25^. Our results indicated that tumor marker sensitivity is too low for oesophageal cancer screening and has poor prognostic significance. The present study also obtained a CA19-9 sensitivity of 42% in gastric cancer, contradicting findings of and Ishigami *et al.*^26^*;* the latter two studies indicated CA19-9 sensitivities of 54.8% and 18%, respectively, in gastric carcinoma. Patai *et al.*^27^ found CA 19-9 is the most useful diagnostic tool to differentiate between pancreatic carcinoma and pancreatitis chronica (both group without cholostasis), as well as for monitoring the patients after surgery of a gastrointestinal cancer. Ishigami *et al.*^26^ found that patients positive for both CEA and CA19-9 had significantly higher frequencies of lymph node metastasis, deeper invasion by the tumor, lower rates of curative resection, and higher rates of hepatic metastasis. The present findings were consistent with the studies by Yamao *et al.*^28^ and Ersan *et al.*^29^ The present study indicated a CA19-9 sensitivity of 26%, which is higher than the values reported by Tohru *et al.*^30^ and Ahbeddou *et al.*^31^ (19.9% and 15%, respectively) in colorectal cancer patients. Tohru *et al.*^30^ observed low sensitivity than our result and concluded that preoperative serum level of CA19-9 is a stronger prognostic factor after curative surgery. Ahbeddou *et al.*^31^ found that high serum level was not correlated to any of the investigated clinicopathological characteristics (age, site, stage, tumor differentiation, lymph node involvement). According to Munck-Wikland *et al.*^32^, the appearance of distant metastases is associated with increased CEA levels in esophageal cancer. In addition, the abnormally high serum CEA levels may reflect the metastatic potential of esophageal cancer cells. A significant relationship (*P*=0.0145) was found between elevated serum CEA levels and distant metastasis, whereas no association was observed between the seropositivity of CA 19-9 and resectability, tumor progression, or patient survival^32^.

The present results agree with those obtained by Zheng *et al.*^33^ and Louhimo *et al.*^34^ These studies and our study supported the conclusion that CA19-9 correlated well with the depth of invasion and Dukes staging and aid in predicting the prognosis of patients. Elevated CA 19-9 is related to poor outcome in colorectal cancer patients.

CA19-9 binds to the endothelial cell surface receptors E-selectin and P-selectin, activated by cytokines. This finding suggests the significance of CA19-9 in the adhesion of cancer cells to endothelial cells, resulting in hematogenous metastasis^35^. CA19-9 in very small amounts may be found in healthy patients. CA19-9 does not cause cancer but rather is a protein shed by tumor cells, making it useful as a tumor marker for monitoring the course of the disease^36^.

In this study, combined analysis of CEA and CA19-9 showed that the sensitivity increased to 42% in patients with esophageal cancer. Turkyilmaz *et al.*^37^ suggested that surgical removal of tumor is currently the only potential treatment for cancer. However, many patients are ineligible for surgery at diagnosis because of lymph node metastasis or distant organ metastasis. Therefore measurement of serum CEA and CA 19-9 levels in all subjects with esophageal cancer is important for detection of possible liver metastasis and pancreatic invasion. The presence of metastasis in patients observed during surgical exploration in which preoperative ultrasonography and computerized tomography had not clearly detected the presence of metastasis; the CEA and CA 19-9 levels in these patients were found to be high. Negligible increases were observed in the serum CEA and/or CA 19-9 levels in patients with non-metastatic cancer. Thus, monitoring the progressive increases of these markers in esophageal cancer patients can predict liver metastasis and pancreatic invasion^37^.

There was increase in sensitivity of combined CEA and CA19-9 (58%) in patients with gastric cancer. The results of the current study were consistent with that obtained by Mattar *et al.*^38^ who found a combined CEA and CA19-9 sensitivity of 43.2% in gastric cancer patients. They observed that combined assay of preoperative serum levels of CEA, CA 19-9, and CA 72-4 has provided additional prognostic information for patients after gastric cancer resection^38^.

Kochi *et al.*^39^ demonstrated that patients with elevated serum CEA levels exhibit a significantly higher risk of having all recurrence factors than patients with normal serum CEA levels. Liver metastasis was identified as the highest risk factor for elevated serum CEA levels. Patients with elevated serum CA19-9 levels are subjected to a significantly higher risk for peritoneal metastases and distant metastases than subjects with normal serum CA19-9 levels. Patients with elevated levels of both markers showed a significantly worse prognosis than patients with normal levels of the two markers. CEA and CA19-9 levels are increased in patients with multiple organ infiltration, advanced lymph node metastasis, peritoneal metastasis, liver metastasis, or other distant metastasis. These findings indicated that the proportions with elevation in these tumor markers increase as the cancer progresses and that levels of combined CEA and CA19-9 provided additional prognostic information in patients with primary gastric cancer^39^.

Our results were contradictory to the results obtained by Tuncer *et al.*^40^, that is, 80% in esophageal cancer and 90% in gastric cancer. They proved that CEA levels are useful in determining relapse and follow-up on responses to the treatment in patients with gastric and esophageal cancers. CA19-9 is also an adhesion molecule expressed on vascular endothelium. The positive detection of CA19-9 shows a correlation with the depth, magnitude, and metastasis of the tumor to various organs and tissues. Both tumor markers show a peak increase in patients with hepatic metastasis. The decrease in hepatic elimination of CEA and CA19-9 is known to significantly influence the elevation of serum levels of tumor markers during hepatic metastasis^40^.

The increase in combined CEA and CA19-9 sensitivity (72%) in patients with colon cancer indicated no significant increase against CEA sensitivity (74%) and was similar to the results of the study by Tsavaris *et al.*^41^.

Duffy *et al.*^23^ evaluated that CEA exhibits 100% sensitivity in detecting metastatic liver disease. The liver is the main site for metastasis from colorectal cancer, with 60% of patients developing metastasis in this organ. The liver appears to be the only site of metastatic disease in 40% of patients who die from colorectal cancer. Approximately 25% of these patients are candidates for hepatic resection, and the 5-year survival for patients who undergo surgery ranges from 21% to 48%. Therefore, hepatic resection is the most successful and currently the only potential treatment for liver metastasis from colorectal cancer. The liver is the primary site for metabolism of CEA. Uptake initially occurs in the Kupffer cells, which modify CEA by removing sialic acid residues. Asialo CEA is then endocytosed by liver parenchymal cells and subsequently degraded. Certain benign liver diseases impair liver function, resulting in CEA clearance. CEA is most useful for the early detection of liver metastasis in patients diagnosed with colorectal cancer. Given the relative success of surgery in resecting hepatic metastases, serial determinations of the marker are recommended for detecting the spread of cancer to the liver^23^.

El-Awady *et al.*^42^ indicated that CEA is a metastatic potentiator. The high serum CEA detected through CRC screening programs should be considered a marker of malignancy, especially in patients with appropriate symptoms, which include diarrhoea or constipation, changes in stool consistency, narrow stools, rectal bleeding or blood in the stool, pain, cramps, or gas in the abdomen, pain during bowel movements, Irritable Bowel Syndrome (IBS), change in bowel habits, continual urges to defecate, weakness or fatigue, unexplained weight loss, shortness of breath, and iron deficiency anaemia. The preoperative CEA in CRC patients identifies subsets with favorable, indolent, and uneven biological behavior. Moreover, the addition of preoperative CEA level to conventional staging forms a strong prognostic tool and supplies an adopted practice guideline initiative for follow-up and therapy in CRC^42^.

*In vitro* studies by Ogata *et al.*^43^ showed that CEA produced by colorectal cancer cells is highly correlated with tumor resistance to lymphokine-activated killer (LAK) cells by inhibiting cytolysis, LAK cell infiltration, and LAK cell adherence to colorectal cancer cells. In experimental models, tumors that produce CEA exhibit a higher rate of metastatic implantation within the liver than non-CEA producers. Although 90% of colorectal cancers produce CEA, elevated serum levels are rarely observed during diagnosis because the CEA enters the portal circulation and undergoes first-pass metabolism by the liver^43^.

Grotowski^44^ concluded that preoperative serum CEA level is useful for the diagnosis and prognosis of recurrence in colorectal cancer patients. The CEA level increases as the cancer progresses. Expression of CA19-9 has been described in various malignancies including colorectal cancer. CA19-9 has not been advocated as a screening test for colorectal cancer. CA19-9 is increased in advanced stages of colorectal cancer^44^. Carpelan-Holmstroma *et al.*^19^ compared the utility of serum CEA, CA19-9, CA242, CA72-4, and human chorionic gonadotropin (hCG) β levels in the follow-up of 102 surgically treated colorectal cancer patients, 40 of which developed clinical recurrence. In patients with recurrent disease, serum samples were obtained during the clinical recurrence, whereas in the disease-free group, serum samples were obtained postoperatively. CEA showed the highest diagnostic accuracy in the detection of recurrent colorectal cancer. Inclusion of CA19-9, CA242, CA72-4, or hCGβ in the model exhibited no improvement in diagnostic accuracy^19^. Thus, CEA is the most useful surveillance marker for patients surgically treated for colorectal cancer.

Thus, in the present study, we concluded that the combination of CEA and CA19-9 exhibits higher diagnostic efficiency than each tumor marker in esophageal and gastric cancer. For these two cancer types, the combination of the results of both markers provides better prediction results and a more accurate clinical picture than either CEA or CA19-9 only. In colon cancer, CEA individually exhibits higher sensitivity than CEA and CA19-9 in combination during initial diagnosis.

Cancer does not develop at once. Environmental factors contribute to the development of many cancers. A cancer patient may have been providing an alterable tumor-coddling milieu. Thus, the answer lies in the prevention and the ultra-early detection of the disease. Optimally, the future of diagnosis and treatment is cure and not 5-year survival. The concentration of tumor markers is primarily determined to monitor the success of treatment procedures (particularly with advanced malignancy). The remaining procedures for treatment assessment, such as X-ray, magnetic resonance imaging, and scanning are considerably costly and involve an increased risk for human health. High levels of CEA and CA19-9 during the initial diagnosis provide greater prognostic significance and can benefit clinical practice. Further advancement in screening, diagnostics, and targeted therapeutics can potentially increase cancer survival rates.
